# Special Issue: “Therapeutic Potential of Natural Compounds: Insights into Mechanisms, Molecular Docking, and Biological Activities”

**DOI:** 10.3390/ijms262311251

**Published:** 2025-11-21

**Authors:** Rafał Hałasa

**Affiliations:** Department of Pharmaceutical Microbiology, Medical University of Gdansk, Al. J. Hallera 107, 80-416 Gdansk, Poland; rafal.halasa@gumed.edu.pl

Since the dawn of time, the treatment of infectious diseases, as well as modern non-communicable diseases, has faced challenges in terms of finding effective drug candidates with minimal side effects. Despite the development of new drugs, many diseases (HIV/AIDS, malaria, hypertension, diabetes, and cancer) continue to cause high mortality in the general population. Currently, many research groups, faced with the challenges associated with global public health, are turning their search to natural products that could provide a source of new innovative drugs [1,2]. This is particularly true for anticancer drugs such as paclitaxel isolated from *Taxus brevifolia* and vinblastine isolated from *Catharanthus roseus*; antimalarial drugs such as quinine isolated from *Cinchona* spp. and artemisinin isolated from *Artemisia annua* [1,2,3,4,5,6,7]; and the antibacterial penicillin derived from *Penicillium* spp. All of these drugs were discovered in natural products and effectively treat diseases. There are many natural compounds that can be isolated from various sources (plants, animals, and microorganisms), but the medicinal properties of plants have been known since time immemorial and have long been used to combat diseases or alleviate symptoms [8,9,10,11,12,13,14]. Plants have evolved to produce numerous molecules that protect them from harmful environmental factors and pests, and these can also provide potential therapeutic effects in human diseases [15,16,17,18,19,20,21]. After a period of dominance of modern medicine, there has been a noticeable rise in the popularity of traditional/folk medicine, as evidenced by the increasing use of medicinal plants in the treatment of diseases [15,16,17,18,19,20,21,22,23,24]. In the United Kingdom, Germany, China, and France [25,26], many extracts derived from medicinal plants are available by prescription. Approximately one-quarter of all drugs approved by the Food and Drug Administration (FDA) and/or the European Medicines Agency (EMA) are of plant origin [27,28]. Additionally, approximately one-third of FDA-approved drugs from the last 20 years have been based on natural products or their derivatives [29,30]. Identifying new drug candidates from natural products is challenging due to their complex composition, typically comprising a mixture of diverse chemical compounds. The biological activity of these extracts is often a result of synergistic interactions between several chemicals that are present [31,32]. Furthermore, one should also take into account the complexity of various diseases, such as degenerative disorders or cancers, where monotherapy is not effective. Drug discovery based on plant extracts should rely on a comprehensive evaluation of all potential compounds. The rapid emergence and refinement of advanced technologies—such as quantum computing, computational biology approaches, molecular profiling techniques, large-scale data analytics, microfluidics, and artificial intelligence—have provided researchers with powerful tools to comprehensively evaluate the therapeutic potential of plant-derived natural products and to elucidate the molecular mechanisms underlying extract activity under physiologically relevant conditions [33,34]. In addition to compounds with synergistic effects, the extract may also contain antagonistic or masking compounds. Several innovative prefractionation and extraction techniques have been developed [35,36] to enhance the recovery of active compounds, including microwave-, ultrasound-, and enzyme-assisted extraction [37]; membrane separation technologies [38,39]; and molecular distillation methods [40,41]. Current guidelines for drug discovery and modern medicine recommend the use of single compounds as drugs, not whole-plant extracts. However, often, the isolation of a “bioactive compound” renders the compound ineffective. In contrast, folk medicine operates on the opposite assumption, suggesting that the synergistic action of multiple constituents provides a superior therapeutic effect. Therefore, it is essential to understand the molecular effects of whole extracts on the human body.

The implementation of Good Manufacturing Practices (GMP) [42] in the herbal medicine industry has facilitated an increase in the number of herbal medicines approved for therapeutic use, with a growing number currently undergoing clinical trials aimed at obtaining FDA approval. Modern drug development based on herbal products, which plays a crucial role in addressing global health challenges, must therefore be supported by innovative technological solutions. The greatest challenges currently lie in the development of innovative methods that enable the precise identification of the individual components of plant extracts, the determination of their specific therapeutic activities, and the optimization of extraction processes to eliminate interfering substances. Research should focus not only on examining individual substances but also on the complex composition of various substances within plant extracts. Research should also include analyses of the effects on, among others, gene expression and the function of proteins involved in metabolic processes, as this knowledge will contribute to the rational design of pharmacologically relevant molecules. Experimental studies should also employ in vivo evaluations in animal models, particularly mice. Developments in microfluidics, computational analysis of large data sets during drug design and testing, machine learning tools, and molecular docking have enabled better design and testing of chemical substances from plant extracts in the drug discovery process [43,44,45,46]. The research articles included in this Special Issue focus on the phytochemical analysis of whole-plant extracts (*Juglans regia* L. extract) and components isolated from various plant extracts (such as aloperine, genistein, flavonoids extracted from *Aurantii Fructus Immaturus*, and yamogenin), as well as on synthetic derivatives of natural compounds (plantaricin-derived peptides, trans-stilbene derivatives, and chalcone derivatives conjugated with 2,4-dichlorobenzenesulfonamide). Also included are studies on the effects of these compounds on viruses, cancer cells, the immune system, and microorganisms, employing the latest experimental methods and analytical techniques. The review articles included constitute a compendium of current knowledge covering recent advances in research on nutraceuticals and isolated compounds derived from plant extracts, including aurapten, cafestol, curcumin, fargeson A, hesperidin, lycopene, oleanolic acid, resveratrol, rutin, ursolic acid, withaferin A, and juglanin.
